# Application of Kalman Filter Model in the Landslide Deformation Forecast

**DOI:** 10.1038/s41598-020-57881-3

**Published:** 2020-01-23

**Authors:** Fumin Lu, Huaien Zeng

**Affiliations:** National Field Observation and Research Station of Landslides in Three Gorges Reservoir Area of Yangtze River, China Three Gorges University, Yichang, 443002 People’s Republic of China

**Keywords:** Environmental sciences, Natural hazards

## Abstract

Nonlinear exponential trend model is linearized into the linear model, then linearized model parameters are regarded as the state vector containing the dynamic noise to erect Kalman filter model based on exponential trend model to predict the deformation of the rock landslide. Deformation observation values of the landslide are regarded as a time series to erect AR(1) model, then model parameters of AR(1) model are regarded as the state vector containing the dynamic noise to erect Kalman filter model based on AR(1) model to predict the deformation of the rock landslide. The deformation of the landslide is regarded as the function of the time, then Taylor series is used to determine the functional relationship between the deformation of the landslide and the time, and Taylor series is spread to erect Kalman filter model based on Taylor series to predict the deformation of the earthy landslide. The deformation of landslides relates to many factors, the rainfall and the temperature influence the deformation of landslides specially, thus Kalman filter model based on multiple factors is erect to predict the deformation of the earthy landslide on the basis of Taylor series. Numerical examples show that the fitting errors and the forecast errors of the four Kalman filter models are little.

## Introduction

The landslide is a common natural disaster, and the disaster caused by the landslide not only harms the production and the life of people, but also greatly destroys the natural resource and the natural environment^1–3^. In order to provide the early warning of the landslide geohazard, it is important to monitor landslides and erect landslide deformation forecast models^4,5^.

Landslide deformation prediction models include mainly grey model^6–12^, time series model^13–21^, neural network model^22–29^ and wavelet transform model^30–33^.

Grey model weakens the randomness of original data by accumulating original data, and can convert complex raw data into time series consistent with the objective law. Grey model has the advantages that the calculation is simple and the forecast accuracy is high in the short time, but grey model is suitable for the situation that original data show the exponential growth^34^. Time seris model is a parametric time domain analysis model. Time series model establishes the corresponding mathematical model for all kinds of dynamic data, and analyses the mathematical model in order to predict the variation tendencey of data, but time series model is suitable for stationary data^35^. Neural network model has featuers of the parallel computation, distributed information storage, adaptive learning. Neural network model has some advantages in the simulation about nonlinear problems^34^, but neural network model presents some local minimum points, and its velocity of the convergence is slow^36^. Wavelet transform model can provide the local characterization of the signal in the time and frequency domain. Wavelet transform model is suitable for processing the non-stationary signal, but wavelet transform model involve the choice of the threshold value, and the choice of the threshold value is very difficult^37^.

The filter means that the best estimator is obtained by means of processing observation data containing errors. In order to obtain some unknown parameters, some observations must be gathered. The observation value is the function of some parameters, and the observation value contains some errors. Our aim is to obtain the estimated value of the unknown parameter by means of the observation value containing errors. Kalman filter equations are obtained by means of the maximum posterior estimation or the minimum variance estimation, and it uses the previous eatimated value or the recent observation value to estimate the current value. Klman filter uses the state to describe the physical system, and uses the state transition to reflect the inherent law of the system change^38^. Kalman filter is a recursive filtering method. Kalman filter estimates the new state estimator on the basis of the state estimator and the observation value at the current time, and can process massive repeated observation data quikly, and can combine the parameter estimation with the forecast^39^, thus it is used widely in many applications, such as navigation, target trcking, control and data processing^39–43^.

This paper establishes four Kalman filter models, i.e. Kalman filter model based on exponential trend model, Kalman filter model based on AR(1) model, Kalman filter model based on the time factor and Taylor series, Kalman filter model based on multiple factors and Taylor series, and these models are used to forecast the deformation of some landslides. Numerical examples show that the fitting effect and forecast effect of these models are good.

## Methods

### Kalman filter model

The state equation and the observation equation of Kalman filter model are^44–47^:

1 $${{\bf{X}}}_{k+1}={{\boldsymbol{\Phi }}}_{k+1,k}{{\bf{X}}}_{k}+{{\boldsymbol{\Omega }}}_{k}$$

2 $${{\bf{L}}}_{k+1}={{\bf{B}}}_{k+1}{{\bf{X}}}_{k+1}+{{\boldsymbol{\Delta }}}_{k+1}$$

where

**X**_*k*_ = the state vector at the time *t*_*k*_

**L**_*k*_ = the observation vector at the time *t*_*k*_

**Φ**_*k*+1, *k*_ = the state transfer matrix at the time *t*_*k*_ to *t*_*k+1*_

**B**_*k*+1_ = the observation matrix at the time *t*_*k+1*_

**Ω**_*k*_ = the dynamic noise at the time *t*_*k*_

**Δ**_*k*_ = the observation noise at the time *t*_*k*_

The random model of Kalman filter model are^44–47^:

$$\begin{array}{rcl}E({{\boldsymbol{\Omega }}}_{k}) & = & 0,\,E({{\boldsymbol{\Delta }}}_{k})\\  & = & 0,\,\mathrm{cov}({{\boldsymbol{\Omega }}}_{k},\,{{\boldsymbol{\Omega }}}_{j})\\  & = & {D}_{{\boldsymbol{\Omega }}}(k){\delta }_{kj},\,\mathrm{cov}({{\boldsymbol{\Delta }}}_{k},\,{{\boldsymbol{\Delta }}}_{j})\\  & = & {D}_{{\boldsymbol{\Delta }}}(k){\delta }_{kj},\,\mathrm{cov}({{\boldsymbol{\Omega }}}_{k},\,{{\boldsymbol{\Delta }}}_{j})\\  & = & 0,\,E({{\bf{X}}}_{0})\\  & =\, & {\mu }_{{\bf{X}}}(0)\\  & = & {\bf{X}}(0/0),\mathrm{var}({{\bf{X}}}_{0})\\  & = & {D}_{{\bf{X}}}(0),\,\mathrm{cov}({{\bf{X}}}_{0},\,{{\boldsymbol{\Omega }}}_{k})\\  & = & 0,\,\mathrm{cov}({{\bf{X}}}_{0},\,{{\boldsymbol{\Delta }}}_{k})\\  & = & 0\end{array}$$

If *j* = *k*, *δ*_*kj*_ =1, if *j* ≠ *k*, *δ*_*kj*_ =0

where

*E*(**Ω**_*k*_) = the mathematical expectation of **Ω**_*k*_

*E*(Δ_*k*_) = the mathematical expectation of **Δ**_*k*_

cov(**Ω**_*k*_, **Ω**_*j*_) = the covariance of **Ω**_*k*_ and **Ω**_*j*_

D_**Ω**_(*k*) = the variance of **Ω**_*k*_

cov(**Δ**_*k*_, **Δ**_*j*_) = the covariance of **Δ**_*k*_ and **Δ**_*j*_

*D*_Δ_(*k*) = the variance of **Δ**_*k*_

cov(**Ω**_*k*_, **Δ**_*j*_) = the covariance of **Ω**_*k*_ and **Δ**_*j*_

*E*(**X**_0_) = the mathematical expectation of **X**_0_

var(**X**_0_) = the variance of **X**_0_

cov(**X**_0_, **Ω**_*k*_) = the covariance of **X**_0_ and **Ω**_*k*_

cov(**X**_0_, **Δ**_*k*_) = the covariance of **X**_0_ and **Δ**_*k*_

On the basis of the state equation and the observation equation and the random model, the solution of Kalman filter equations are obtained^44–47^:

3 $${\bf{X}}(k/k)={\bf{X}}(k/k-1)+{{\bf{J}}}_{k}[{{\bf{L}}}_{k}-{{\bf{B}}}_{k}{\bf{X}}(k/k-1)]$$

4 $${{D}}_{{\bf{X}}}(k/k)=[{\bf{I}}-{{\bf{J}}}_{k}{{\bf{B}}}_{k}]{{D}}_{{\bf{X}}}(k/k-1)$$

where **I** is a unit matrix, and

5 $${\bf{X}}(k/k-1)={{\boldsymbol{\Phi }}}_{k,k-1}{\bf{X}}(k-1/k-1)$$

6 $${D}_{{\bf{X}}}(k/k-1)={{\boldsymbol{\Phi }}}_{k,k-1}{{D}}_{{\bf{X}}}(k-1/k-1){{\boldsymbol{\Phi }}}_{k,k-1}^{T}+{D}_{{\boldsymbol{\Omega }}}(k-1)$$

7 $${{\bf{J}}}_{k}={D}_{{\bf{X}}}(k/k-1){{\bf{B}}}_{k}^{T}{[{{\bf{B}}}_{k}{D}_{{\bf{X}}}(k/k-1){{\bf{B}}}_{k}^{T}+{D}_{{\boldsymbol{\Delta }}}(k)]}^{-1}$$

### Kalman filter model based on exponential trend model

Wang used exponential trend model to analyze the deformation of the rock landslide^48^. Exponential trend model is a nonlinear model as follows:

8 $$y=a{e}^{bt}$$

where

*a* = the parameter of the model

*b* = the parameter of the model

*t* = the observation time of the model

*y* = the fitting value of the model

Equation (8) can be written as follows:

9 $$\mathrm{ln}\,y=\,\mathrm{ln}\,a+bt$$

Denoting

$$y^{\prime} =\,\mathrm{ln}\,y,\,a^{\prime} =\,\mathrm{ln}\,a$$

Equation (9) can be rewritten as follows:

10 $$y^{\prime} =a^{\prime} +bt$$

On the basis of deformation observation data, *a*′ and b can be obtained by means of least square method, then a can be obtained by means of *a*′.

In order to improve the fitting precision of exponential trend model, we can regard *a*′ and b of Eq. (10) as the state vector containing the dynamic noise to erect Kalman filter model, and then we can obtain following equation:

11 $${y^{\prime} }_{k}={a^{\prime} }_{k}+{b}_{k}{t}_{k}+{\Delta }_{k}$$

where

$a{^{\prime} }_{\kappa }$ = the parameters of the linearized exponential trend model

*b*_*k*_ = the parameters of the linearized exponential trend model

*t*_*k*_ = the observation time

${y^{\prime} }_{k}$ = the natural logarithm of the deformation observation value

Δ_*k*_ = the observation noise at the observation time *t*_*k*_

Denoting

$${{\bf{L}}}_{k}={y^{\prime} }_{k},\,{{\bf{B}}}_{k}=[1\,{t}_{k}],\,{{\bf{X}}}_{k}=\left[\begin{array}{c}{a^{\prime} }_{k}\\ {b}_{k}\end{array}\right],\,{{\boldsymbol{\Delta }}}_{k}={\Delta }_{k}$$

Equation (11) can be expressed as follows:

12 $${{\bf{L}}}_{k}={{\bf{B}}}_{k}{{\bf{X}}}_{k}+{{\boldsymbol{\Delta }}}_{k}$$

For the next time *t*_*k*+1_, Eq. (12) can be written as follows:

13 $${{\bf{L}}}_{k+1}={{\bf{B}}}_{k+1}{{\bf{X}}}_{k+1}+{{\boldsymbol{\Delta }}}_{k+1}$$

In order to make Kalman filter, ***X***_*k*_ is regarded as the state vector containing the dynamic noise, thus the following equation is obtained:

14 $${{\bf{X}}}_{k+1}={{\bf{X}}}_{k}+{{\boldsymbol{\Omega }}}_{k}$$

Equation (14) can be written as follows:

15 $${{\bf{X}}}_{k+1}={{\boldsymbol{\Phi }}}_{k+1,k}{{\bf{X}}}_{k}+{{\boldsymbol{\Omega }}}_{k}$$

where **Φ**_*k*+1, *k*_ = **I**, i.e. **Φ**_*k*+1,*k*_ is a unit matrix.

On the basis of the state Eq. (15) and the observation Eq. (13), by means of Kalman filter equation, Kalman filter can be performed.

### Kalman filter model based on AR(1) model

The deformation value of the landslide at the time *t*_*k*_ can be regarded as a time series^49^ {*y*_*k*_}, thus AR(n) model of the time series is:

16 $${y}_{k}={\varphi }_{1}{y}_{k-1}+{\varphi }_{2}{y}_{k-2}+\cdot \cdot \cdot +{\varphi }_{n}{y}_{k-n}+{a}_{k}$$

In Eq. (16) let *k* = *n* + 1, *n* + 2, …, *N*, we have:

17 $$\left\{\begin{array}{l}{y}_{n+1}={\varphi }_{1}{y}_{n}+{\varphi }_{2}{y}_{n-1}+\cdot \cdot \cdot +{\varphi }_{n}{y}_{1}+{a}_{n+1}\\ {y}_{n+2}={\varphi }_{1}{y}_{n+1}+{\varphi }_{2}{y}_{n}+\cdot \cdot \cdot {\varphi }_{n}{y}_{2}+{a}_{n+2}\\ \cdot \cdot \cdot \cdot \cdot \cdot \cdot \cdot \cdot \cdot \cdot \cdot \cdot \cdot \cdot \cdot \cdot \cdot \cdot \cdot \cdot \cdot \cdot \cdot \cdot \cdot \cdot \cdot \cdot \cdot \cdot \cdot \cdot \cdot \cdot \cdot \cdot \cdot \cdot \\ {y}_{N}={\varphi }_{1}{y}_{N-1}+{\varphi }_{2}{y}_{N-2}+\cdot \cdot \cdot +{\varphi }_{n}{y}_{N-n}+{a}_{N}\end{array}\right.$$

In Eq. (17) let *n* = 1, we have AR(1) model:

18 $$\left\{\begin{array}{l}{y}_{2}={\varphi }_{1}{y}_{1}+{a}_{2}\\ {y}_{3}={\varphi }_{1}{y}_{2}+{a}_{3}\\ \cdot \cdot \cdot \cdot \cdot \cdot \cdot \cdot \cdot \cdot \cdot \cdot \cdot \cdot \\ {y}_{N}={\varphi }_{1}{y}_{N-1}+{a}_{N}\end{array}\right.$$

Denoting

$${{\bf{X}}}_{k+1}={[\begin{array}{cccc}{\varphi }_{1} & {\varphi }_{2} & \mathrm{..}. & {\varphi }_{n}\end{array}]}^{T},{{\bf{B}}}_{k+1}=[\begin{array}{cccc}{y}_{k-1} & {y}_{k-2} & \mathrm{..}. & {y}_{k-n}\end{array}],{{\boldsymbol{\Delta }}}_{k+1}={a}_{k},{{\bf{L}}}_{k+1}={y}_{k}$$

Equation (16) can be rewritten as follows:

19 $${{\bf{L}}}_{k+1}={{\bf{B}}}_{k+1}{{\bf{X}}}_{k+1}+{{\boldsymbol{\Delta }}}_{k+1}$$

To stationary random sequence AR(1), we have:

20 $${{\bf{X}}}_{k+1}={{\bf{X}}}_{k}+{{\boldsymbol{\Omega }}}_{k}$$

Equation (20) can be written as follows:

21 $${{\bf{X}}}_{k+1}={{\boldsymbol{\Phi }}}_{k+1,k}{{\bf{X}}}_{k}+{{\boldsymbol{\Omega }}}_{k}$$

where

$${{\boldsymbol{\Phi }}}_{k+1,k}={\bf{I}}$$

Obviously **X**_*k*_ and **Ω**_*k*_ are the one-dimensional vector to AR(1) model. On the basis of the state Eq. (21) and the observation Eq. (19), by means of Kalman filter equation, Kalman filter can be performed.

### Kalman filter model based on the time factor and Taylor series

The deformation of the landslide can be regarded as the function of the time, because the time interval of the deformation observation to the landslide is very short, and the variation of the deformation value is very small, thus the deformation value of the landslide *x*(*t*_*k*+1_) at the time *t*_*k*+1_ can be spread at the time *t*_*k*_ by means of Taylor series:

22 $$x\left({t}_{k+1}\right)=x\left({t}_{k}\right)+{\left(\frac{\partial x}{\partial t}\right)}_{{t}_{k}}\left({t}_{k+1}-{t}_{k}\right)+\frac{1}{2}{\left(\frac{{\partial }^{2}x}{\partial {t}^{2}}\right)}_{{t}_{k}}{\left({t}_{k+1}-{t}_{k}\right)}^{2}+\frac{1}{6}{\left(\frac{{\partial }^{3}x}{\partial {t}^{3}}\right)}_{{t}_{k}}{\left({t}_{k+1}-{t}_{k}\right)}^{3}+{g}_{k}$$

Denoting

$${x}_{k}=x\left({t}_{k}\right),\,{v}_{k}={\left(\frac{\partial x}{\partial t}\right)}_{{t}_{k}},\,{a}_{k}={\left(\frac{{\partial }^{2}x}{\partial {t}^{2}}\right)}_{{t}_{k}},\,{s}_{k}=\frac{1}{6}{\left(\frac{{\partial }^{3}x}{\partial {t}^{3}}\right)}_{{t}_{k}}$$

Equation (22) can be rewritten as follows:

23 $${x}_{k+1}={x}_{k}+{v}_{k}({t}_{k+1}-{t}_{k})+\frac{1}{2}{a}_{k}{({t}_{k+1}-{t}_{k})}^{2}+{s}_{k}{({t}_{k+1}-{t}_{k})}^{3}+{g}_{k}$$

where

*v*_*k*_ = the deformation velocity at the time *t*_*k*_

*a*_*k*_ = the deformation acceleration at the time *t*_*k*_

*s*_*k*_ = the influence of the third power of the time variation to the deformation

*g*_*k*_ = the remainder term of Taylor series

Because *g*_*k*_ is very small, it can be regarded as the dynamic noise whose mathematical expectation is 0. Let

24 $${v}_{k+1}={v}_{k}+{a}_{k}({t}_{k+1}-{t}_{k})+{c}_{k}$$

25 $${a}_{k+1}={a}_{k}+{r}_{k}$$

26 $${s}_{k+1}={s}_{k}+{p}_{k}$$

where

*c*_*k*_ = the small perturbation

*r*_*k*_ = the small perturbation

*p*_*k*_ = the small perturbation

They can be regarded as the dynamic noises whose mathematical expectation are 0.

Equations (23) to (26) can be written in the matrix form as:

27 $$\left[\begin{array}{c}{x}_{k+1}\\ {v}_{k+1}\\ {a}_{k+1}\\ {s}_{k+1}\end{array}\right]=\left[\begin{array}{cccc}1 & ({t}_{k+1}-{t}_{k}) & \frac{1}{2}{({t}_{k+1}-{t}_{k})}^{2} & {({t}_{k+1}-{t}_{k})}^{3}\\ 0 & 1 & ({t}_{k+1}-{t}_{k}) & 0\\ 0 & 0 & 1 & 0\\ 0 & 0 & 0 & 1\end{array}\right]\left[\begin{array}{c}{x}_{k}\\ {v}_{k}\\ {a}_{k}\\ {s}_{k}\end{array}\right]+\left[\begin{array}{c}{g}_{k}\\ {c}_{k}\\ {r}_{k}\\ {p}_{k}\end{array}\right]$$

Denoting

$${{\bf{X}}}_{k+1}=\left[\begin{array}{c}{x}_{k+1}\\ {v}_{k+1}\\ {a}_{k+1}\\ {s}_{k+1}\end{array}\right],\,{{\boldsymbol{\Phi }}}_{k+1,k}=\left[\begin{array}{cccc}1 & ({t}_{k+1}-{t}_{k}) & \frac{1}{2}{({t}_{k+1}-{t}_{k})}^{2} & {({t}_{k+1}-{t}_{k})}^{3}\\ 0 & 1 & ({t}_{k+1}-{t}_{k}) & 0\\ 0 & 0 & 1 & 0\\ 0 & 0 & 0 & 1\end{array}\right],\,{{\boldsymbol{\Omega }}}_{k}=\left[\begin{array}{c}{g}_{k}\\ {c}_{k}\\ {r}_{k}\\ {p}_{k}\end{array}\right]$$

Equation (27) can be written as follows:

28 $${{\bf{X}}}_{k+1}={{\boldsymbol{\Phi }}}_{k+1,k}{{\bf{X}}}_{k}+{{\boldsymbol{\Omega }}}_{k}$$

Equation (28) is the state equation of Kalman filter model.

To the deformation observation, the following Equation is obtained:

29 $${l}_{k+1}={x}_{k+1}+{\Delta }_{k+1}$$

Denoting

$${{\bf{L}}}_{k+1}={l}_{k+1},\,{{\bf{B}}}_{k+1}=[\begin{array}{cccc}1 & 0 & 0 & 0\end{array}],\,{{\boldsymbol{\Delta }}}_{k+1}={\Delta }_{k+1}$$

Equation (29) can be be rewritten as follows:

30 $${{\bf{L}}}_{k+1}={{\bf{B}}}_{k+1}{{\bf{X}}}_{k+1}+{{\boldsymbol{\Delta }}}_{k+1}$$

Equation (30) is the observation equation of Kalman filter model.

On the basis of the state Eq. (28) and the observation Eq. (30), by means of Kalman filter equation, Kalman filter can be performed.

### Kalman filter model based on multiple factors and Taylor series

The deformation of landslides relates to many factors, the rainfall and temperature influence the deformation of landslides specially. The rainfall causes the runoff of the slope, and the rainwater infiltrates into the landslide mass to increases the weight of the landslide mass, thus the sliding power of the landside mass is augmented. The change of the temperature causes the constriction or the dilation of the crack of the landslide and influences the stability of the landslide. Thus the deformation of the landslide can be regarded as the function of the time and the rainfall of every month and the temperature, i.e.

31 $${F}_{x}=x(t,\,j,\,w)$$

where

*t* = the observation time

*j* = the rainfall of every month at t

*w* = the temperature at t

*F*_*x*_ = the deformation of the landslide

Because the time interval of the deformation observation to the landslide is very short, and the variation of the deformation value is very small, thus the deformation value *x*(*t*_*k*+1_, *j*_*k*+1_, *w*_*k*+1_) at *t*_*k*+1_ can be expanded by means of Taylor series, i.e.

32 $$\begin{array}{lll}x({t}_{k+1},{j}_{k+1},{w}_{k+1}) & = & x({t}_{k},{j}_{k},{w}_{k})+{(\frac{{\rm{\partial }}x}{{\rm{\partial }}t})}_{{t}_{k}}({t}_{k+1}-{t}_{k})\\  &  & +\frac{1}{2}{(\frac{{{\rm{\partial }}}^{2}x}{{\rm{\partial }}{t}^{2}})}_{{t}_{k}}{({t}_{k+1}-{t}_{k})}^{2}+{(\frac{{\rm{\partial }}x}{{\rm{\partial }}j})}_{{j}_{k}}({j}_{k+1}-{j}_{k})+\frac{1}{2}{(\frac{{{\rm{\partial }}}^{2}x}{{\rm{\partial }}{j}^{2}})}_{{j}_{k}}{({j}_{k+1}-{j}_{k})}^{2}\\  &  & +{(\frac{{\rm{\partial }}x}{{\rm{\partial }}w})}_{{w}_{k}}({w}_{k+1}-{w}_{k})+\frac{1}{2}{(\frac{{{\rm{\partial }}}^{2}x}{{\rm{\partial }}{w}^{2}})}_{{w}_{k}}{({w}_{k+1}-{w}_{k})}^{2}+{g}_{k}\end{array}$$

Denoting

$${v}_{k}={\left(\frac{\partial x}{\partial t}\right)}_{{t}_{k}},\,{a}_{k}={\left(\frac{{\partial }^{2}x}{\partial {t}^{2}}\right)}_{{t}_{k}},\,{b}_{k}={\left(\frac{\partial x}{\partial j}\right)}_{{j}_{k}},\,{c}_{k}={\left(\frac{{\partial }^{2}x}{\partial {j}^{2}}\right)}_{{j}_{k}},\,{s}_{k}={\left(\frac{\partial x}{\partial w}\right)}_{{w}_{k}},\,{y}_{k}={\left(\frac{{\partial }^{2}x}{\partial {w}^{2}}\right)}_{{w}_{k}},\,{x}_{k}=x({t}_{k},{j}_{k},{w}_{k})$$

Equation (32) can be rewritten as follows:

33 $$\begin{array}{rcl}{x}_{k+1} & = & {x}_{k}+{v}_{k}({t}_{k+1}-{t}_{k})+\frac{1}{2}{a}_{k}{({t}_{k+1}-{t}_{k})}^{2}+{b}_{k}({j}_{k+1}-{j}_{k})\\  &  & +\frac{1}{2}{c}_{k}{({j}_{k+1}-{j}_{k})}^{2}+{s}_{k}({w}_{k+1}-{w}_{k})+\frac{1}{2}{y}_{k}{({w}_{k+1}-{w}_{k})}^{2}+{g}_{k}\end{array}$$

where

*g*_*k*_ = the remainder term of Taylor series

Because *g*_*k*_ is very small, it can be regarded as the dynamic noise whose mathematical expectation is 0, Let

34 $${v}_{k+1}={v}_{k}+{a}_{k}({t}_{k+1}-{t}_{k})+{d}_{k}$$

35 $${a}_{k+1}={a}_{k}+{r}_{k}$$

36 $${b}_{k+1}={b}_{k}+{c}_{k}({j}_{k+1}-{j}_{k})+{p}_{k}$$

37 $${c}_{k+1}={c}_{k}+{e}_{k}$$

38 $${s}_{k+1}={s}_{k}+{y}_{k}({w}_{k+1}-{w}_{k})+{h}_{k}$$

39 $${y}_{k+1}={y}_{k}+{z}_{k}$$

where

*d*_*k*_ = the very small perturbation

*r*_*k*_ = the very small perturbation

*p*_*k*_ = the very small perturbation

*e*_*k*_ = the very small perturbation

*h*_*k*_ = the very small perturbation

*z*_*k*_ = the very small perturbation

They can be regarded as the dynamic noises whose mathematical expectation are 0.

Equations (33) to (39) can be written in the matrix form as:

40 $$\begin{array}{c}\left[\begin{array}{c}{x}_{k+1}\\ {v}_{k+1}\\ {a}_{k+1}\\ {b}_{k+1}\\ {c}_{k+1}\\ {s}_{k+1}\\ {y}_{k+1}\end{array}\right]=\left[\begin{array}{ccccccc}1 & {t}_{k+1}-{t}_{k} & \frac{1}{2}{({t}_{k+1}-{t}_{k})}^{2} & {j}_{k+1}-{j}_{k} & \frac{1}{2}{({j}_{k+1}-{j}_{k})}^{2} & {w}_{k+1}-{w}_{k} & \frac{1}{2}{({w}_{k+1}-{w}_{k})}^{2}\\ 0 & 1 & {t}_{k+1}-{t}_{k} & 0 & 0 & 0 & 0\\ 0 & 0 & 1 & 0 & 0 & 0 & 0\\ 0 & 0 & 0 & 1 & {j}_{k+1}-{j}_{k} & 0 & 0\\ 0 & 0 & 0 & 0 & 1 & 0 & 0\\ 0 & 0 & 0 & 0 & 0 & 1 & {w}_{k+1}-{w}_{k}\\ 0 & 0 & 0 & 0 & 0 & 0 & 1\end{array}\right]\left[\begin{array}{c}{x}_{k}\\ {v}_{k}\\ {a}_{k}\\ {b}_{k}\\ {c}_{k}\\ {s}_{k}\\ {y}_{k}\end{array}\right]\\ +{\left[\begin{array}{ccccccc}{g}_{k} & {d}_{k} & {r}_{k} & {p}_{k} & {e}_{k} & {h}_{k} & {z}_{k}\end{array}\right]}^{T}\end{array}$$

Denoting

$${{\bf{X}}}_{k}={[\begin{array}{ccccccc}{x}_{k} & {v}_{k} & {a}_{k} & {b}_{k} & {c}_{k} & {s}_{k} & {y}_{k}\end{array}]}^{T},{{\boldsymbol{\Omega }}}_{k}={[\begin{array}{ccccccc}{g}_{k} & {d}_{k} & {r}_{k} & {p}_{k} & {e}_{k} & {h}_{k} & {z}_{k}\end{array}]}^{T}$$

$${{\boldsymbol{\Phi }}}_{k+1,k}=\left[\begin{array}{ccccccc}1 & {t}_{k+1}-{t}_{k} & \frac{1}{2}{({t}_{k+1}-{t}_{k})}^{2} & {j}_{k+1}-{j}_{k} & \frac{1}{2}{({j}_{k+1}-{j}_{k})}^{2} & {w}_{k+1}-{w}_{k} & \frac{1}{2}{({w}_{k+1}-{w}_{k})}^{2}\\ 0 & 1 & {t}_{k+1}-{t}_{k} & 0 & 0 & 0 & 0\\ 0 & 0 & 1 & 0 & 0 & 0 & 0\\ 0 & 0 & 0 & 1 & {j}_{k+1}-{j}_{k} & 0 & 0\\ 0 & 0 & 0 & 0 & 1 & 0 & 0\\ 0 & 0 & 0 & 0 & 0 & 1 & {w}_{k+1}-{w}_{k}\\ 0 & 0 & 0 & 0 & 0 & 0 & 1\end{array}\right]$$

Equation (40) can be written as follows:

41 $${{\bf{X}}}_{k+1}={{\boldsymbol{\Phi }}}_{k+1,k}{{\bf{X}}}_{k}+{{\boldsymbol{\Omega }}}_{k}$$

Equation (41) is the state equation of Kalman filter model.

To the deformation observation, we have

42 $${l}_{k+1}={x}_{k+1}+{\Delta }_{k+1}$$

where

*l*_*k*+1_ = the deformation observation value at the time *t*_*k*+1_

Δ_*k*+1_ = is the observation noise at the time *t*_*k*+1_

Denoting

$${{\bf{L}}}_{k+1}={l}_{k+1},{{\bf{B}}}_{k+1}=[\begin{array}{ccccccc}1 & 0 & 0 & 0 & 0 & 0 & 0\end{array}]$$

Equation (42) can be rewritten as follows:

43 $${{\bf{L}}}_{k+1}={{\bf{B}}}_{k+1}{{\bf{X}}}_{k+1}+{{\boldsymbol{\Delta }}}_{k+1}$$

On the basis of the state Eq. (41) and the observation Eq. (43), by means of Kalman filter equation, Kalman filter can be performed.

## Results and Discussion

### The example of the calculation about Kalman filter model based on exponential trend model

We choose horizontal displacement observation data of the monitoring point *F*_A_ in a rock landslide to perform some calculations, initial values are set as follows:

$$\begin{array}{rcl}{{\bf{D}}}_{{\boldsymbol{\Delta }}}(k) & = & \pm 1\,{\rm{mm}},{\bf{X}}(0/0)=\left[\begin{array}{c}2.88932\\ 0.12862\end{array}\right],\\ {{\bf{D}}}_{{\bf{X}}}(0/0) & = & \left[\begin{array}{cc}1 & 0\\ 0 & 1\end{array}\right],\\ {{\bf{D}}}_{{\boldsymbol{\Omega }}}(k) & = & \left[\begin{array}{cc}1 & 0\\ 0 & 1\end{array}\right]\end{array}$$

Where

**D**_**Δ**_(*k*) = the variance of horizontal displacement observation values

**X**(0/0) = the calculation value of parameters of exponential trend model

Some calculation results are listed in Table 1.

**Table 1:** Horizontal displacement observation values and their filter values of the monitoring point *F*_A_.

Observation time (year-month)	Observation value (mm)	Exponential trend model		Kalman filter model	
		Fitted value (mm)	Residual (mm)	Fitted value (mm)	Residual (mm)
2007–12	10.32	20.45	10.13	13.45	3.13
2008–12	26.96	23.26	−3.70	25.60	−1.36
2009–12	34.07	26.45	−7.62	34.43	0.36
2010–12	38.65	30.08	−8.57	39.17	0.52
2011–12	42.98	34.21	−8.77	43.32	0.34
2012–12	44.93	38.90	−6.03	45.20	0.27
2013–12	47.16	44.24	−2.92	47.33	0.17
2014–12	48.38	50.31	1.93	48.52	0.14
2015–12	49.95	57.22	7.27	50.04	0.09
2016–12	51.75	65.07	13.32	51.82	0.07

Table 1 shows that residuals of exponential trend model are greater than 1 mm, the maximum residual is 13.32 mm, and the minimum residual is 1.93 mm. Residuals of Kalman filter model are less, two residuals are greater than 1 mm, and other residuals are less than 1 mm.

Exponential trend model predicts that the horizontal displacement value of the monitoring point *F*_A_ in December 2017 is 74.01 mm, the horizontal displacement observation value of the monitoring point *F*_A_ in December 2017 was 52.50 mm, the forecast error is 21.51 mm. Kalman filter model predicts that the horizontal displacement value of the monitoring point *F*_A_ in December 2017 is 53.27 mm, the forecast error is 0.77 mm. Computed results show that Kalman filter model based on exponential trend model is better than exponential trend model in the fitting precision and the prediction precision.

Parameters of exponential trend model are fixed values, thus the ability that the model adapts to the observation data is weaked, and the fitting precision and the prediction precision of the model are reduced.

Kalman filter model based on exponential trend model regard parameters of exponential trend model as the state vector containing the dynamic noise to carry out Kalman filter. In the process of Kalman filter, parameters of the model constantly change, thus the ability that the model adapts to the observation data is enhanced, and the fitting precision and forecast precision of the model are improved.

### The example of the calculation about Kalman filter model based on AR(1) model

We choose vertical displacement observation data of the monitoring point *P*_10_ in a rock landslide to perform some calculations, and initial values are set as follows:

$$\begin{array}{rcl}{{\bf{D}}}_{{\boldsymbol{\Delta }}}(k) & = & \pm 1.0\,{\rm{mm}},\\ {\bf{X}}(0/0) & = & 35.8,\\ {{\bf{D}}}_{{\bf{X}}}(0/0) & = & 1,{{\bf{D}}}_{\Omega }(k)=1\end{array}$$

where

**D**_**Δ**_(*k*) = the variance of vertical displacement observation values

35.8 = the vertical displacement observation value of the monitoring point *P*_10_ in December 2015

Some calculation results are listed in Table 2.

**Table 2:** Vertical displacement observation values and their filter values of the monitoring point *P*_10_.

Observation time (year-month)	Observation value (mm)	AR(1) model		Kalman filter model	
		Fitted value (mm)	Residual (mm)	Fitted value (mm)	Residual (mm)
2016–01	36.4	35.21	−1.19	36.29	−0.11
2016–02	37.2	36.43	−0.77	37.27	0.07
2016–03	37.6	37.32	−0.28	37.60	0.00
2016–04	38.3	38.11	−0.19	38.28	−0.02
2016–05	38.5	38.61	0.11	38.50	0.00
2016–06	39.7	39.52	−0.18	39.73	0.03
2016–07	40.2	40.33	0.13	40.21	0.01
2016–08	41.6	41.52	−0.08	41.60	0.00
2016–09	42.0	42.17	0.17	42.02	0.02
2016–10	42.5	42.42	−0.08	42.51	0.01
2016–11	42.9	43.18	0.28	42.92	0.02
2016–12	43.7	43.56	−0.14	43.70	0.00
2017–01	43.9	43.99	0.09	43.89	−0.01
2017–02	44.2	44.15	−0.05	44.22	0.02
2017–03	44.6	44.69	0.09	44.57	−0.03
2017–04	45.1	45.00	−0.10	45.11	0.01
2017–05	45.9	45.28	−0.62	45.91	0.01
2017–06	46.7	46.93	0.23	46.70	0.00
2017–07	48.0	47.11	−0.89	48.03	0.03
2017–08	48.8	48.22	−0.58	48.76	−0.04
2017–09	49.2	49.33	0.13	49.21	0.01
2017–10	49.7	49.64	−0.06	49.70	0.00
2017–11	50.3	50.77	0.47	50.28	−0.02

Table 2 shows that residuals of AR(1) model are greater, the maximum residual is −1.19 mm, the minimum residual is −0.05 mm. Residuals of Kalman filter model are less, the maximum residual is −0.11 mm, the minimum residual is 0.00 mm.

AR(1) model predicts that the vertical displacement value of the monitoring point *P*_10_ in December 2017 is 50.71 mm, the vertical displacement observation value of the monitoring point *P*_10_ in December 2017 was 50.9 mm, the forecast error is 0.19 mm. Kalman filter model predicts that the vertical displacement value of the monitoring point *P*_10_ in December 2017 is 50.88 mm, the forecast error is 0.02 mm. Computed results show that Kalman filter model based on AR(1) model is better than AR(1) model in the fitting precision and the prediction precision.

The parameters of AR(1) model is a fixed value, thus the ability that the model adapts to the observation data is weaked, and the fitting precision and the prediction precision of the model are reduced.

Kalman filter model based on AR(1) model regard the parameters of AR(1) model as the state vector containing the dynamic noise to carry out Kalman filter. In the process of Kalman filter, the parameter of the model constantly change, thus the ability that the model adapts to the observation data is enhanced, and the fitting precision and forecast precision of the model are improved.

### The example of the calculation about Kalman filter model based on the time factor and Taylor serie

We choose horizontal displacement observation data of the monitoring point *G*_8_ in a earthy landslide to perform some calculations, initial values are set as follows:

$$\begin{array}{rcl}{{\bf{D}}}_{{\boldsymbol{\Delta }}}(k) & = & \pm 1\,{\rm{mm}},{\bf{X}}(0/0)=\left[\begin{array}{c}448.5\\ 0\\ 0\\ 0\end{array}\right],\\ {{\bf{D}}}_{{\bf{X}}}(0/0) & = & \left[\begin{array}{cccc}1 & 0 & 0 & 0\\ 0 & 1 & 0 & 0\\ 0 & 0 & 1 & 0\\ 0 & 0 & 0 & 1\end{array}\right],\\ {{\bf{D}}}_{{\boldsymbol{\Omega }}}(k) & = & \left[\begin{array}{cccc}1 & 0 & 0 & 0\\ 0 & 1 & 0 & 0\\ 0 & 0 & 1 & 0\\ 0 & 0 & 0 & 1\end{array}\right]\end{array}$$

where

**D**_**Δ**_(*k*) = the variance of horizontal displacement observation values

448.5 = the horizontal displacement observation value of the monitoring point *G*_8_ on December 10, 2016

Some calculation results are listed in Table 3.

**Table 3:** Horizontal displacement observation values and their filter values of the monitoring point *G*_8_.

Observation time (year-month-day)	Observation value (mm)	Fitted value (mm)	Residual (mm)
2017–01–12	468.6	468.3	−0.3
2017–02–20	471.5	471.3	−0.2
2017–03–18	487.1	486.0	−1.1
2017–04–18	498.9	499.3	0.4
2017–05–18	525.3	524.6	−0.7
2017–06–16	551.3	551.1	−0.2
2017–07–16	577.1	577.8	0.7
2017–08–17	594.3	595.6	1.3
2017–09–19	623.0	622.3	−0.7
2017–10–17	624.3	625.2	0.9
2017–11–11	639.0	638.1	−0.9

Table 3 shows that residuals obtained by Kalman filter method are less, the maximum residual is 1.3 mm, the minimum residual is −0.2 mm, two residuals are greater than 1 mm, other residuals are less than 1 mm. Some residuals are negative, and others are positive. It shows that residuals are random and fitting errors are less.

Kalman filter model predicts that the horizontal displacement value of the monitoring point *G*_8_ on December 8, 2017 is 648.9 mm, the horizontal displacement observation value of the monitoring point *G*_8_ on December 8, 2017 is 650.6 mm, the forecast error is 1.7 mm, thus the forecast error is very small.

Because the state vector *X*_*k*_ of Kalman filter model based on the time factor and Taylor series constantly change in the process of Kalman filter, the ability that the model adapts to the observation data is enhanced, and the fitting precision and forecast precision of the model are improved.

### The example of the calculation about Kalman filter model based on multiple factors and Taylor series

A landslide is a earthy, some cracks pass through the landslide, the rainfall and the temperature cause the deformation of the landslide. We use the horizontal displacement observation data of the monitoring point *H*_4_ at the landslide to perform some calculations, initial values are set as follows:

$$\begin{array}{rcl}{{\bf{D}}}_{{\boldsymbol{\Delta }}}(k) & = & \pm 1\,{\rm{mm}},\\ {\bf{X}}(0/0) & = & [\begin{array}{ccccccc}115.4 & 0 & 0 & 0 & 0 & 0 & 0\end{array}]\end{array}$$

$${{\bf{D}}}_{{\bf{X}}}(0/0)=\left[\begin{array}{ccccccc}1 & 0 & 0 & 0 & 0 & 0 & 0\\ 0 & 1 & 0 & 0 & 0 & 0 & 0\\ 0 & 0 & 1 & 0 & 0 & 0 & 0\\ 0 & 0 & 0 & 1 & 0 & 0 & 0\\ 0 & 0 & 0 & 0 & 1 & 0 & 0\\ 0 & 0 & 0 & 0 & 0 & 1 & 0\\ 0 & 0 & 0 & 0 & 0 & 0 & 1\end{array}\right],{{\bf{D}}}_{\Omega }(k)=\left[\begin{array}{ccccccc}1 & 0 & 0 & 0 & 0 & 0 & 0\\ 0 & 1 & 0 & 0 & 0 & 0 & 0\\ 0 & 0 & 1 & 0 & 0 & 0 & 0\\ 0 & 0 & 0 & 1 & 0 & 0 & 0\\ 0 & 0 & 0 & 0 & 1 & 0 & 0\\ 0 & 0 & 0 & 0 & 0 & 1 & 0\\ 0 & 0 & 0 & 0 & 0 & 0 & 1\end{array}\right]$$

where

**D**_**Δ**_(*k*) = the variance of horizontal displacement observation values

115.4 = the horizontal displacement observation value of the monitoring point *H*_4_ on March 15, 2016.

Some calculation results are listed in Table 4.

**Table 4:** Horizontal displacement observation values and their filter values of the monitoring point *H*_4_.

Observation time (year-month-day)	Rainfall of month (mm)	Temperature (°C)	Observation value (mm)	Residual 1 (mm)	Residual 2 (mm)
2016–04–19	49.5	18.2	118.3	33.30	0.56
2016–05–19	173.0	24.6	128.2	19.48	0.07
2016–06–18	124.6	26.3	172.7	15.49	−0.92
2016–07–20	187.9	30.4	228.4	−22.21	0.84
2016–08–18	114.9	27.4	253.9	−23.93	0.95
2016–09–21	121.4	24.1	259.7	−29.09	−0.02
2016–10–18	46.0	18.9	290.3	−36.90	−0.78
2016–11–18	49.3	13.8	294.6	−35.41	−0.21
2016–12–14	8.6	8.5	298.3	−12.99	−0.36
2017–01–15	6.6	7.2	296.0	6.49	0.81
2017–02–25	20.8	9.8	299.3	16.16	−0.57
2017–03–19	40.5	14.4	298.0	24.22	0.99
2017–04–18	76.0	17.8	304.9	24.50	0.21
2017–05–16	129.4	22.8	310.9	27.60	0.75
2017–06–15	86.9	25.6	338.7	38.51	−0.11
2017–07–14	162.6	29.5	364.4	19.57	0.75
2017–08–16	198.7	28.2	380.9	−3.41	0.98
2017–09–07	44.3	25.8	438.8	−11.28	0.87
2017–10–13	102.0	19.2	449.9	−50.38	−0.51

*Residual 1 is the residual of quadratic polynomial regression model, Residual 2 is the residual of Kalman filter model.

Table 4 shows that when rainfall and temperature increase, the deformation of the monitoring point *H*_4_ increases, thus the deformatin of the landslide relates to the rainfall and the temperature. Residuals of quadratic polynomial regression model are greater than 3 mm, the maximum residual is −50.38 mm, the minimum residual is −3.14 mm, fitting errors of quadratic polynomial regression model are great, while residuals of Kalman filter model based on multiple factors are less than 1 mm, and the maximum residual is 0.99 mm, the minimum residual is −0.02 mm, the fitting effect of Kalman filter model based on multiple factors is good.

Quadratic polynomial regression model predicts that the horizontal displacement value of the monitoring point *H*_4_ on November 15, 2017 is 431.84 mm, the horizontal displacement observation value of the monitoring point *H*_4_ on November 15, 2017 is 454.9 mm, the forecast error is 23.06 mm. Kalman filter model based on multiple factors predicts that the horizontal displacement value of the monitoring point *H*_4_ on November 15, 2017 is 456.20 mm, the forecast error is 1.30 mm. Computed results show that Kalman filter model based on multiple factors is better than quadratic polynomial regression model in the fitting precision and the prediction precision.

The parameters of quadratic polynomial regression model is a fixed value, thus the ability that the model adapts to the observation data is weaked, and the fitting precision and the prediction precision of the model are reduced.

Because the state vector *X*_*k*_ of Kalman filter model based on multiple factors and Taylor series series constantly change in the process of Kalman filter, the ability that the model adapts to the observation data is enhanced, and the fitting precision and forecast precision of the model are improved.

This paper establishes four Kalman filter models, i.e. Kalman filter model based on exponential trend model, Kalman filter model based on AR(1) model, Kalman filter model based on the time factor and Taylor series, Kalman filter model based on multiple factors and Taylor series, and these models are used to forecast the deformation of some landslides.

Nonlinear exponential trend model is linearized into the linear model, then linearized model parameters are regarded as the state vector containing the dynamic noise to erect Kalman filter model based on exponential trend model, and computed results show that Kalman filter model based on exponential trend model is better than exponential trend model in the fitting precision and the prediction precision. The model parameters of AR(1) model are regarded as the state vector containing the dynamic noise to erect Kalman filter model based on AR(1) model, and computed results show that Kalman filter model based on AR(1) model is better than AR(1) model in the fitting precision and the prediction precision. Taylor series is used to establish the functional relationship between the deformation of the landslide and the time, then Kalman filter model based on Taylor series is established, and computed results show that Kalman filter model based on Taylor series is goog in the fitting precision and the prediction precision. The deformation of landslides relates to many factors, the effect of the atmospheric rainfall and the air temperature to the deformation of the landslide is significant, thus the deformation of the landslide is regarded as the function of the time and the rainfall of every month and the temperature to establish Kalman filter model based on multiple factors, and computed results show that Kalman filter model based on multiple factors is better than quadratic polynomial regression model in the fitting precision and the prediction precision.

Because the state vector of Kalman filter model constantly change in the process of Kalman filter, the ability that the model adapts to the observation data is enhanced, thus the fitting precision and prediction precision of these Kalman filter model are improved.
